# Prevalence and factors influencing the distribution of influenza viruses in Kenya: Seven-year hospital-based surveillance of influenza-like illness (2007–2013)

**DOI:** 10.1371/journal.pone.0237857

**Published:** 2020-08-21

**Authors:** Therese Umuhoza, Wallace D. Bulimo, Julius Oyugi, David Schnabel, James D. Mancuso

**Affiliations:** 1 Institute of Tropical and Infectious Diseases, University of Nairobi, Nairobi, Kenya; 2 Department of Emerging Infectious Diseases, United State Army Medical Research Directorate – Africa, Nairobi, Kenya; 3 Department of Biochemistry, School of Medicine, University of Nairobi, Nairobi, Kenya; 4 US President’s Malaria Initiative, Freetown, Sierra Leone; 5 Department of Preventive Medicine and Biostatistics, Uniformed Services University of the Health Sciences, Bethesda, Maryland, United States of America; The Scripps Research Institute, UNITED STATES

## Abstract

**Background:**

Influenza viruses remain a global threat with the potential to trigger outbreaks and pandemics. Globally, seasonal influenza viruses’ mortality range from 291 243–645 832 annually, of which 17% occurs in Sub-Saharan Africa. We sought to estimate the overall prevalence of influenza infections in Kenya, identifying factors influencing the distribution of these infections, and describe trends in occurrence from 2007 to 2013.

**Methods:**

Surveillance was conducted at eight district hospital sites countrywide. Participants who met the case definition for influenza-like illness were enrolled in the surveillance program. The nasopharyngeal specimens were collected from all participants. We tested all specimens for influenza viruses with quantitative reverse transcriptase real-time polymerase chain reaction (RT-qPCR) assay. Bivariate and multivariate log-binomial regression was performed with a statistically significant level of p<0.005. An administrative map of Kenya was used to locate the geographical distribution of surveillance sites in counties. We visualized the monthly trend of influenza viruses with a graph and chart using exponential smoothing at a damping factor of 0.5 over the study period (2007–2013).

**Results:**

A total of 17446 participants enrolled in the program. The overall prevalence of influenza viruses was 19% (n = 3230), of which 76% (n = 2449) were type A, 21% (n = 669) type B and 3% (n = 112) A/ B coinfection. Of those with type A, 59% (n = 1451) were not subtyped. Seasonal influenza A/H3N2 was found in 48% (n = 475), influenza A/H1N1/pdm 2009 in 43% (n = 434), and seasonal influenza A/ H1N1 in 9% (n = 88) participants. Both genders were represented, whereas a large proportion of participants 55% were ≤1year age. Influenza prevalence was high, 2 times more in other age categories compared to ≤1year age. Category of occupation other than children and school attendees had a high prevalence of influenza virus (p< <0.001). The monthly trends of influenza viruses’ positivity showed no seasonal pattern. Influenza types A and B co-circulated throughout the annual calendar during seven years of the surveillance.

**Conclusions:**

Influenza viruses circulate year-round and occur among children as well as the adult population in Kenya. Occupational and school-based settings showed a higher prevalence of influenza viruses. There were no regular seasonal patterns for influenza viruses.

## Introduction

Influenza viruses cause a significant global burden with the potential to trigger devastating outbreaks and or pandemics [1]. Seasonal influenza viruses cause global mortality ranging from 291 243–645 832 individuals annually, 17% of which occurs in Sub-Saharan Africa [2]. To prevent, detect, and respond to the global threat of influenza, the World Health Organization (WHO) established a Global Influenza Surveillance and Response System (GISRS) in 1952 [3, 4]. GISRS monitors the evolution of influenza viruses to provide recommendations for laboratory diagnostics, vaccines formulations, antiviral susceptibility, and risk assessment. The WHO GISRS also serves as a global alert system for the emergence of influenza viruses with pandemic potential. In response to the GISRS network, several nations and partners have progressively initiated an influenza surveillance program to detect influenza virus activities and developed control measures [3].

In the African region, national influenza surveillance programs increased from 21 to 127 for Influenza-like Illness (ILIs) and 2 to 98 for Severe Acute Respiratory Illness (SARI) since 2006 in response to the 2005 international health regulations (IHR) [5]. Before 2006, no comprehensive surveillance of influenza or other viral respiratory illnesses was being undertaken in Kenya. With the emergent pandemic threat due to the then little known virulent avian influenza caused by the highly pathogenic influenza A (H5N1) virus in 2003 [6], the United States Department of Defense’s (US DoD) Global Emerging Infections Surveillance and Response System (GEIS) expanded its outreach in respiratory virus surveillance by initiating influenza surveillance to outside continental US (OCONUS) DoD laboratories, including the United States Army Medical Research Unit-Kenya (USAMRU-K) [7]. This was a strategic Force Health Protection response by US DoD to support military readiness by anticipating major health threats to service members in the event of a military operation in this strategic area.

The primary objective of the respiratory surveillance program was to monitor the emergence and characterize the epidemiology and clinical significance of respiratory pathogens with special emphasis on influenza viruses, focusing on civilian and military populations. The specific objective was to identify changes in circulating influenza virus subtypes and genotype strains which may impact disease severity, transmissibility, and treatment and prevention effectiveness across time and geography.

Data from this surveillance program have local and regional benefits. Locally, this endeavor supports the Kenya Ministry of Health (MoH) public health surveillance, one of the most important functions carried out by the MoH. Through disease surveillance programs, the MoH can prioritize and implement public health interventions in a timely fashion. This program established an influenza surveillance network in Kenya with links between the USAMRU-K, Kenya Defence Forces (KDF), Kenya Medical Research Institute (KEMRI), MoH and WHO. Furthermore, during the period that followed the initiation of surveillance activities, the KEMRI Influenza laboratory gained the required skills and experience through the above partnerships to become a national and regional reference laboratory in 2009. This surveillance system may also provide global benefit through early warning of the circulation of new and dangerous influenza subtypes and local benefits in the event of influenza epidemics. This surveillance system enhanced pandemic preparedness and reporting capabilities required by the 2005 International Health Regulations (IHR) [8]. At the same time, other sentinel influenza systems were also put in place by the MoH with technical support from the Centers for Disease Control and Prevention-Kenya (CDC-K) [9]. These two systems were designed to be complementary, with the DoD system focused on surveillance at district hospitals (sometimes called sub-county hospitals after Kenya government devolution in 2010) and CDC-K surveillance on provincial hospitals (now called county hospitals). The combination of these two systems was expected to give a robust, geographically-representative assessment of the epidemiology of respiratory viruses in Kenya, including the burden of disease, risk factors, trends, and circulating strains.

The influenza surveillance from Kenya’s provincial hospitals between 2007 and 2013 has been reported previously [9]. Here, utilizing ILI data collected at district hospitals over roughly the same time period between 2007 and 2013, we complete the picture of influenza virus surveillance in Kenya by estimating the overall prevalence of infection, factors influencing the distribution of infections, and trends of influenza viruses in the country.

## Methods

### Study sites and population

**Fig 1:**
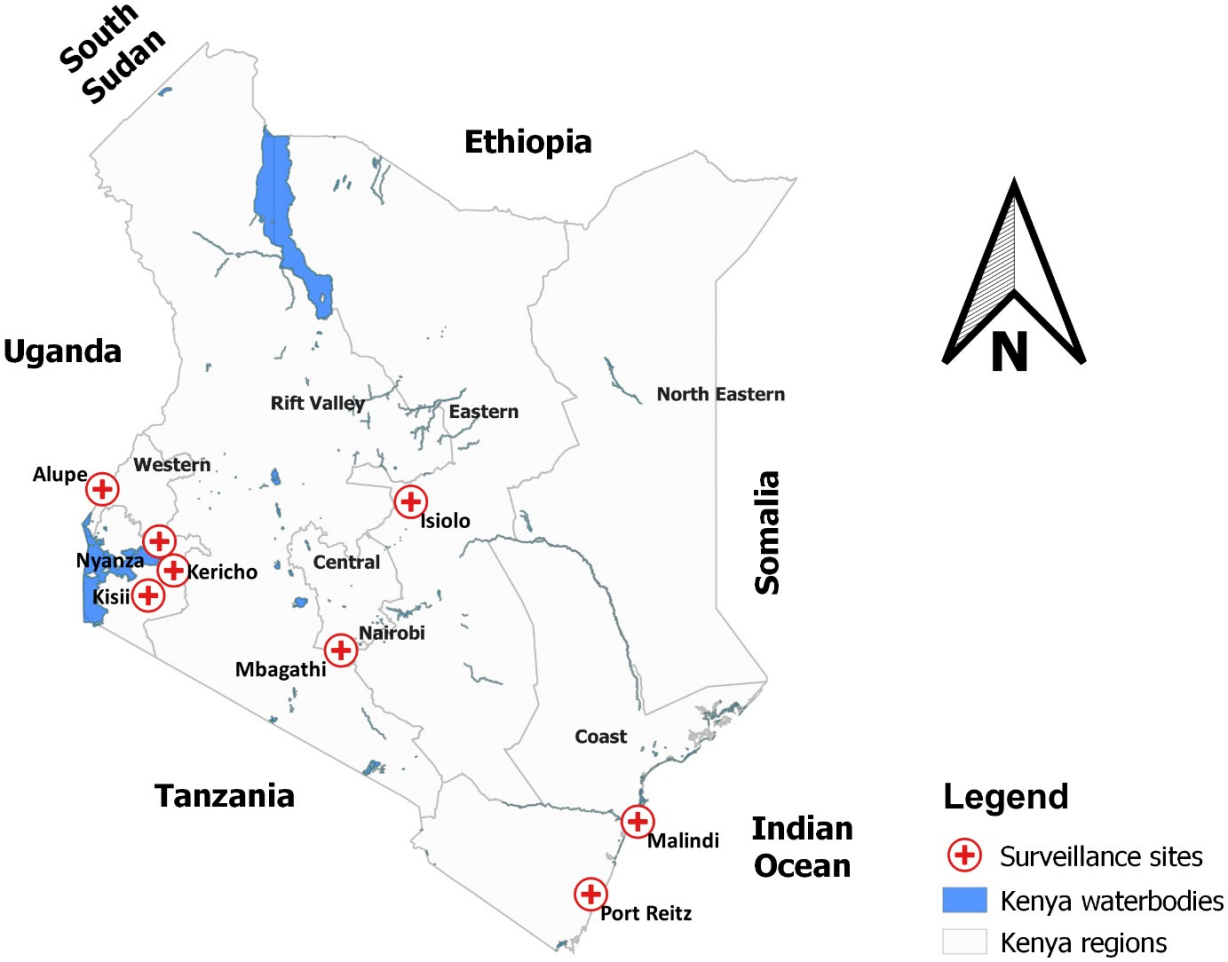
Influenza and other respiratory viruses program surveillance sites.

The joint USAMRU-K and KEMRI protocol for influenza virus surveillance included eight surveillance sites (Fig 1). The study population included all ILI participants for whom the submitted nasopharyngeal specimens met quality control standards in the surveillance program during the period from January 2007 to December 2013 at these sites. Participants included all age groups (infants, children, adults, and elderly) where possible, both gender (male and female) were represented. Surveillance sites were selected accounting for regional population variation, frequency of international movement through the site, and each regional variation in respiratory disease incidence and reporting, and the security situation in the region. The protocol was implemented at several sites including one level five referral hospital (New Nyanza provincial hospital in Kisumu), six district hospitals (Mbagathi district hospital in Nairobi, Malindi district hospital in Kilifi, Port Reitz district hospital in Mombasa, district hospital in Isiolo, district hospital in Kisii, and district hospital in Kericho), and one sub-county hospital (Alupe sub-district hospital in Busia).

A study-specific clinical officer was assigned to each surveillance site. Prior to commencing the study, clinical officers were employed and trained to conduct surveillance for ILI according to the Walter Reed Army Institute of Research (WRAIR) and the KEMRI Institutional Review Boards (IRBs) approved ethical principles under protocol numbers WRAIR#1267 and SSC#981 respectively. Participants with ILI were recruited under the program between January 2007 and December 2013. Participants in the outpatient settings were enrolled if they consented and met the ILI case definition. The informed written consent was obtained from each participant either in English, Kiswahili, or a local language, and the written consent for minors was provided by parents or guardians. Participants were recruited during normal working hours specifically on Mondays, Wednesdays, and Fridays. At each surveillance site, a maximum of five participants was eligible for recruitment per day. A questionnaire was administered to all participants to record information including demographics (age, sex, occupation, village, workplace, residence, and travel history), signs & symptoms, and animal exposure.

### Case definition

The study adopted the WHO case definition for influenza-like illness (ILI) [10]. This was defined as any individual presenting in outpatient services at the surveillance site with 1) fever >38°C (oral or equivalent), 2) cough and 3) onset of illness within the previous 10 days.

### Specimen processing

The nasopharyngeal specimens were taken using Dacron swabs and placed in 1 ml of Viral Transport Medium (VTM) in a cryovial and immediately stored a dry shipper under liquid nitrogen vapors. In cases where liquid nitrogen was not accessible immediately, the specimens were kept at +4°C and transported to the laboratory within 48 hours, to be frozen at -80°C to maintain the cold chain for virus integrity. The cryovials containing the specimens were labeled with unique study numbers using permanent marker pens. The study numbers were assigned by the clinical officer at each surveillance site, who maintained a log of all patients and their study numbers. The study number was the only identifier on the questionnaires and entered in the computerized database established at USAMRU-K. USAMRU-K personnel oversaw the logistics of sample collection, transportation, and accession to the central KEMRI Influenza Laboratory in Nairobi.

### Laboratory testing

Detection of influenza viruses in the patient specimens was performed using the quantitative reverse transcriptase real-time polymerase chain reaction (RT-qPCR) assay. The primer and probe sequences for influenza viruses were provided by the CDC. Viral RNA was extracted from patient specimens using the QIAGEN extraction kits following the manufacturer’s protocols (QIAGEN GmbH, Hilden, Germany). One-step RT-PCR was performed using the Applied Biosystems 7500Fast instrument (Applied Biosystems Inc; Foster City, CA USA). The initial step for the assay involved a reverse transcription step for 30 minutes at 48°C followed by transcriptase inactivation for 10 minutes at 95°C. The thermo-cycling conditions comprised of 45 cycles of denaturation step for 15 seconds at 95°C, followed by primer annealing at 50°C and template extension for one minute at 68°C. All runs were performed together with appropriate controls. The results were interpreted based on cycle threshold (Ct) values in reference to positive and negative controls. Any influenza A and B with Ct value of <40 were considered positive and those with Ct value of ≥ 40 were deemed negative. Samples that tested positive for influenza A were further subtyped for H3N2, H1N1, H5N1, and pandemic H1N1/pdm 2009 using subtype-specific oligonucleotide primers.

### Patient data management

All demographic data from the eight surveillance sites were collected using a standard paper questionnaire. Questionnaires and samples were assessed for quality by USAMRU-K laboratory and data managers. The nasopharyngeal samples which met quality control standards were entered into the laboratory management logbooks. Data retrieved from questionnaires were entered into a project-specific Microsoft Access database.

### Data analysis

The outcomes of influenza-like illness (ILI) laboratory testing was confirmed for the presence of influenza virus (positive) or absence of influenza virus (negative). The prevalence was expressed as the proportion of positive influenza virus in the total ILI tested population, and the estimate was described by participants’ demographic factors and clinical symptoms. The target population included all ILI participants in the surveillance program for the period of 2007 to 2013. We used exact 95% confidence intervals (CI) and chi-square test to measure the differences in demographic variable categories. The log-binomial regression model was performed by bivariate and multivariate to measure the association of predictor variables (demographic and clinical) and the outcome of interest (presence or absence of influenza viruses) with a report of prevalence ratios. We adjusted predictor variables in the multivariate model with a significance level of p<0.05 to account for confounding factors. Two-Way interaction was assessed by adding a hashtag symbol (#) in the model where necessary. Finally, the model was selected by the Bayesian Information Criterion (BIC), the lower BIC indicated the best-fitted model. The analysis was performed with STATA^®^13 (STATA Corporation, College Station, TX, USA). The exponential smoothing with a damping factor of 0.5 was used to visualize the trends over time.

### Ethical consideration

Before commencing study activities, the study was reviewed and approved by the Walter Reed Army Institute of Research (WRAIR) Institutional Review Board (IRB) and the Kenya Medical Research Institute (KEMRI) Scientific and Ethics Review Unit (SERU) under protocol numbers WRAIR#1267 and KEMRI/SERU SSC#981 respectively.

## Results

Of the eight surveillance sites, 17,446 participants met the enrolment and inclusion criteria. The proportion of participants testing positive for influenza viruses were (19%, N = 3,230), and 81% (N = 14,216) were negative for influenza viruses (Table 1). The demographic characteristics of participants indicated both genders were represented with (52%, N = 9,116) male and (48%, N = 8330) female. A large proportion of participants (55%, N = 9695) were children below one year old (≤1year). The median age in the study population was of 1 year, ranging from 2 months to 75 years. The prevalence of influenza viruses was different in the age categories. Those in the age category of 5 to ≤ 18years had a high prevalence (33%), and as were those in the 19–49 years category (33%). Other age categories including 2–4 years and ≤1year. These two age categories had a proportion of influenza virus (23%) and (13%) respectively. Participants aged ≥ 50 years were represented less, although 21% of these had tested positive for influenza virus. Differences in influenza virus proportions were observed for other demographic factors, including attend school, sick contacts, occupation, and exposure to animals (Table 1). Besides, the influenza proportions varied significantly according to geography and time. There was no substantial difference in the prevalence of influenza viruses for gender categories or residential areas.

**Table 1:** Demographic characteristics of the study participants by influenza virus status.

Variable/	Influenza Positive	Influenza Negative	Total Population	Chi-square*
Outcome	n (%)	n (%)	N (%)	P-value
Overall	3230(19%)	14216(81)	17446	
**Gender**				**0.792**
Male	1681 (18)	7435(82)	9116(52)	
Female	1549(19)	6781(81)	8330(48)	
Age*				**<0.0001**
≤1 year	1296(13)	8399 (87)	9695(55)	
2 to 4 years	1424(23)	4787 (77)	6211(35)	
5 to ≤ 18 years	397(33)	801(67)	1198(6)	
19–49 years	110(33)	218(67)	328 (2)	
50+ years	3 (21)	11(79)	14 (0.08)	
**Occupation**				**<0.0001**
Children	346(33)	709(67)	1055 (6)	
Other*	2882(18)	13505(82)	16387(94)	
**Residence**				**0.977**
Urban	3170(19)	13953(81)	17123 (98)	
Rural	60(19)	263 (81)	323 (2)	
**Sick contact**				**0.016**
Yes	1187(19)	4906(81)	6093(35)	
No	2042(18)	9307(82)	11349(65)	
**Attends school**				**<0.0001**
Yes	794 (24)	2464(76)	3258(19)	
No	2435 (17)	11745(83)	14180 (81)	
Birds exposure*				**0.022**
Yes	1344(18)	6231(82)	7575(43)	
No	1886(19)	7985(81)	9871(57)	
**Pigs exposure**				**<0.0001**
Yes	93 (12)	660(88)	753(4)	
No	3137(19)	13556(81)	16693(96)	
**Cats exposure**				**0.051**
Yes	610(20)	2478(80)	3088(18)	
No	2620(18)	11738(82)	14358(82)	
**Location**				**<0.0001**
Alupe	140(7)	1741(93)	1881(11)	
Isiolo	259(19)	1085(81)	1344(7)	
Kericho	371(19)	1621(81)	1992(11)	
Kisii	638(22)	2311(78)	2949(17)	
Malindi	249(19)	1077(81)	1326(8)	
Mbagathi	547(19)	2262(81)	2809(16)	
Nyanza	631(21)	2307(79)	2938(17)	
Port Reitz	377(19)	1629(81)	2006(11)	
**Year**				**<0.0001**
2007	812(28)	2113(72)	2925(17)	
2008	517(17)	2535(83)	3052(17)	
2009	883(23)	2918(77)	3801(22)	
2010	352(12)	2690(88)	3042(17)	
2011	385(17)	1894(83)	2279(13)	
2012	167(12)	1172(88)	1339(8)	
2013	114(11)	894(89)	1008(6)	

Age categories * ≤1 year (infant), 2 to 4 years (Toddler), 5 to ≤ 18 years (Child to Adolescence), 19–49 years (Adult), and 50+ years (Senior Adult). Birds exposure* any domesticated bird. Chi-square* P-value indicates the difference in influenza virus proportion in variable categories. * The category of occupation that included several adult professionals (student, military, other)

**Fig 2:**
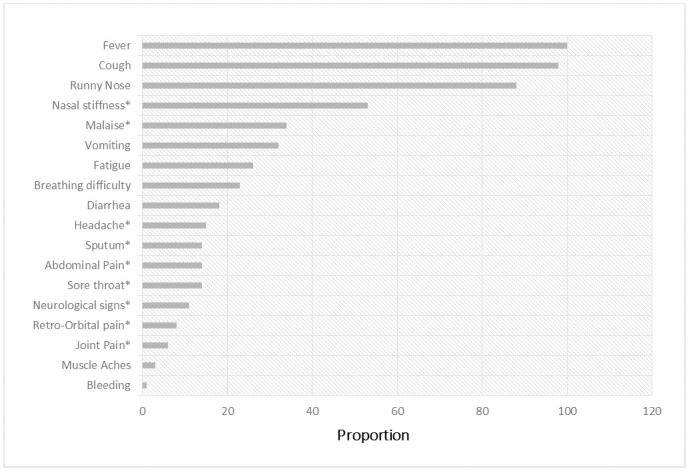
Clinical characteristics of the study participants with influenza-like illness (ILI). *Missing values >10%.

Amongst the ILI participants, clinical symptoms varied. Thus, fever (100%), cough (98%), and runny nose (88%) were the most prevalent symptoms (Fig 2). Other clinical characteristics included (53%) nasal stuffiness, (34%) malaise, (32%) vomiting, (26%) fatigue, and (23%) difficulty of breathing. Less than (20%) of participants reported diarrhea, headache, sore throat, sputum, abdominal pain, retro-orbital pain, joint pain, muscle aches, bleeding, and neurological signs.

A crude proportion of clinical symptoms with influenza virus are described in annex table (S1 Table). Since cough and fever were captured in the case definition, we adjusted other clinical symptoms with age categories. The result indicated, only difficult breathing was positively associated with influenza virus prevalence. The prevalence of influenza viruses was less likely in participants without difficulty breathing (PR = 0.68; 95% CI [0.55–0.83], p<0.0001) compared to participants with difficulty breathing.

The overall prevalence of influenza viruses during the study period was 19% (n = 3,230). Influenza virus type A was the most common accounting for 76% (n = 2,449), and type B had 21% (n = 669) proportion. Coinfection of subtype A and B were identified in 3% (n = 112) participants. Of those with type A, 59% (n = 1451) were not subtyped and 41% (n = 998) were subtyped. Seasonal A/H3N2 was found in 48% (n = 475), A (H1N1) pdm 2009 in 43% (n = 434), and seasonal A/ H1N1 in 9% (n = 88), and there was one case with a coinfection of strains (A/H3N2) and (A/H1N1) pdm 2009.

For each demographic factor, crude and adjusted prevalence ratios (PRs) of influenza viruses were reported and displayed in Table 2. Both the crude and adjusted models indicated that age categories had a significant association with influenza virus prevalence. The prevalence of influenza was 1.66 times high in 2–4 years old (toddlers) compared to ≤1year (infants). Those of 5 to ≤ 18 years old (children-adolescence) had a higher prevalence of influenza virus 2.2 times more than ≤1year (infants). Influenza virus prevalence was also high 1.9 times in 19–49 years old (adult) compared to ≤1year (infants). Influenza virus prevalence was high 1.2 times in other occupations compared to children category. Those who didn’t attend school had a less prevalence of influenza virus, 0.7 lower than school attended participants.

**Table 2:** Prevalence ratio of influenza viruses in ILI patients, Kenya (2006–2013).

Variable	Crude PR*	P-Value	Adjusted PR*	P-Value
Outcome	(95% CI)		(95%CI)	
Gender		0.79		
Male	Ref			
Female	1.00(0.947–1.07)			
**Age**		**<0.0001**		
≤1 year	Ref		Ref	
2 to 4 years	1.71(1.60–1.83)	<0.0001	1.66(1.55–1.78)	<0.0001
5 to ≤ 18 years	2.47(2.25–2.72)	<0.0001	2.23(2.02–2.46)	<0.0001
19–49 years	2.50(2.13–2.94)	<0.0001	1.93(1.59–2.36)	<0.0001
50+ years	1.60(0.58–4.37)	0.357	1.28(0.47–3.47)	0.628
**Occupation**		**<0.0001**		
Children	Ref		Ref	
Other	1.86(1.69–2.04)		1.26(1.12–1.41)	<0.0001
**Sick contact**		**0.016**		
Yes	Ref		Ref	
No	0.92(0.86–0.98)		0.95(0.89–1.02)	0.185
**Attends school**		**<0.0001**		
Yes	Ref		Ref	
No	0.70(0.65–0.75)		0.76(0.71–0.82)	<0.0001
**Birds exposure**		**0.022**		
Yes	Ref		Ref	
No	1.07(1.01–1.14)		1.06(0.99–1.13)	0.066
**Pigs exposure**		**<0.0001**		
Yes	Ref		Ref	
No	1.52(1.25–1.84)		1.36(1.12–1.64)	0.002
**Cats exposure**		**0.049**		
Yes	Ref		Ref	
No	0.92(0.85–0.99)		0.90(0.83–0.98)	0.023

*PR = Prevalence ratio, CI = Confidence interval Age categories * ≤1 year (infant), 2 to 4 years (Toddler), 5 to ≤ 18 years (Child to Adolescence), 19–49 years (Adult) and 50+ years (Senior Adult). Birds exposure* any domesticated bird. Chi-square* P-value indicates the difference in influenza virus proportion in variable categories. * The category of occupation that included several adult professionals (student, military, other).

**Fig 3:**
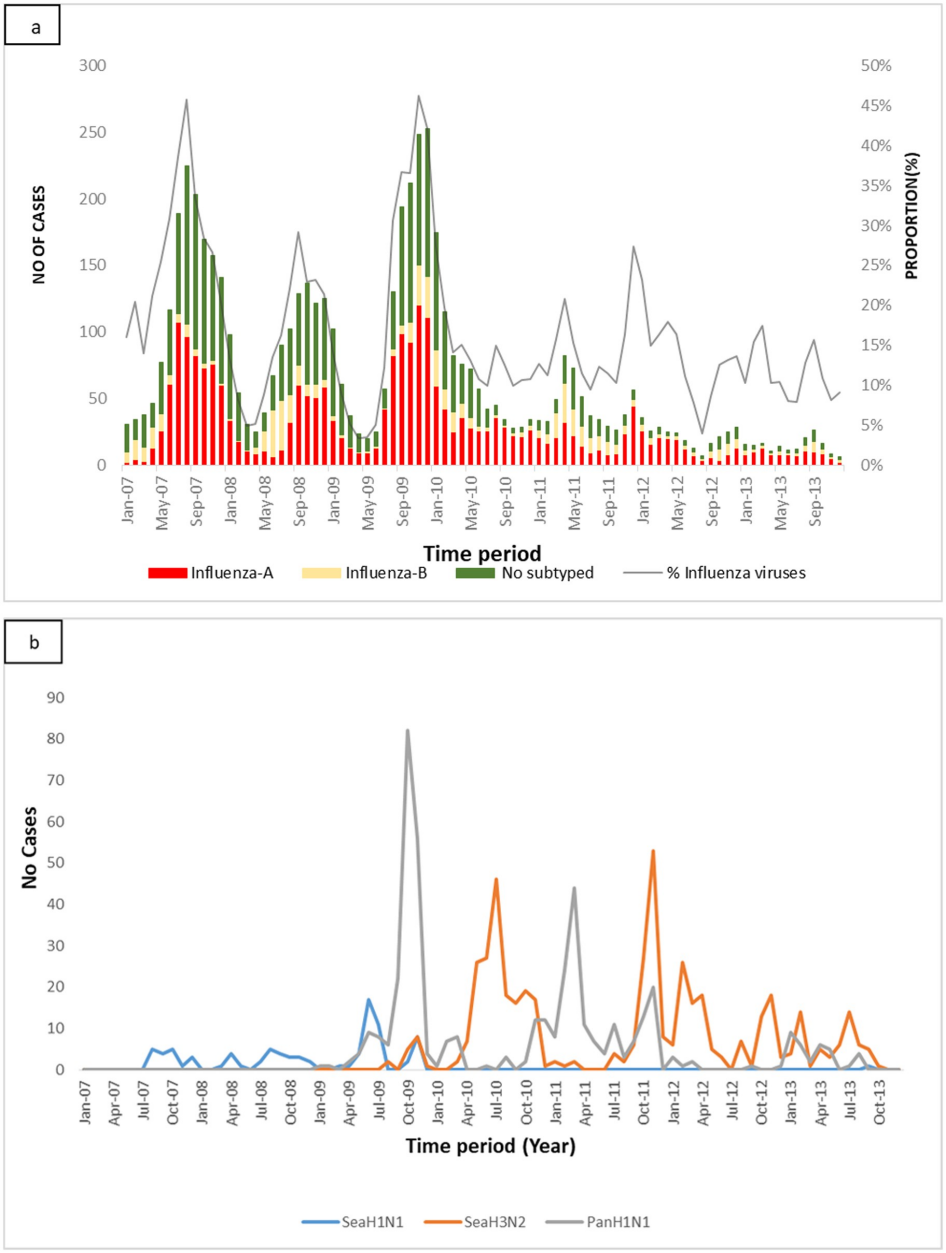
**a**. Influenza viruses monthly trends by Influenza-like Illness (ILI) surveillance program, Kenya. **b**. Influenza virus A subtypes monthly trends by Influenza-like Illness (ILI) surveillance program, Kenya.

Influenza viruses were found circulating at all surveillance sites (Table 1). The prevalence of influenza showed variability over seven years of the surveillance program. There was year-round influenza viral activity evident in Kenya. We recorded two major spikes in influenza prevalence in May-September, 2007, and September-December 2009 (Fig 3a). Both influenza subtypes A and B co-circulated throughout the year during the seven-year surveillance program. Although some variability existed over the interval, subtype A was generally the most dominant. Seasonal influenza A/H1N1 was seen before the emerging of Influenza A (H1N1)pdm 2009 (Fig 3b). Thereafter, influenza A/seasonal H3N2 was noted to co-circulate with A/2009pandemic H1N1. During the study period, two major influenza outbreaks occurred in Kenya. The first one was due to influenza B in a school in the western region of Kenya in 2006–2007 followed by the 2009–2010 countrywide outbreak, which was part of the pandemic caused by the A/H1N1/pdm09 strain.

## Discussion

In this report, among the patients with influenza-like illness (ILI) at the eight surveillance sites in Kenya, 19% were found to have been infected with influenza viruses. The prevalence of influenza infections was slightly higher than the 15% published in the previous study of ILI in provincial hospitals in Kenya [9].

Whereas a greater proportion of influenza virus was noted during the initial years of the surveillance program, i.e. 2007, and in 2009. The proportion of influenza viruses varied over time and location. Those proportions could be attributed to a greater risk of circulating influenza viruses during those initial years, where there were more compliance and enthusiasm in the recruitment than in subsequent years. However, the higher proportion could also be due to differential recruitment and selection procedures applied during the initial years of the study. As this surveillance system contributed to the early detection and warning of the A/H1N1/pdm 2009 occurrence in Kenya, the proportion for that year is believed to be due to an increased risk of influenza. Influenza A/seasonal H3N2 was generally more prevalent (48%) throughout the study period. Nevertheless, in the early years of the study period when few samples were subtyped, the seasonal A/ H1N1 subtype predominated. The emergence of subtype A/H1N1/pdm 2009 resulted in complete displacement and replacement of seasonal A/H1N1 [11]. This is similar to what was observed in other places in the southern hemisphere during the period of 2009 Influenza pandemic [12]. Similar to other southern hemisphere countries, the seasonal periodicity of influenza virus infections (April-September) was evident in years of greater influenza burden than other years.

The prevalence of influenza virus was high (33%) in the children to adolescence age categories as well as the adult category. Those proportions were similar to the published proportion of influenza-positive (34%) in the older children group in a study conducted among 15 African countries [5, 13]. The results differed slightly in other regions of the world including China, India, Colombia, and Peru [14–18]. The difference in prevalence could be explained by the disparities in geography, population diversity, diagnostic methods used, and sampling methods in general. In this study, it was observed that the majority of the study participants were infants (≤1year) and toddlers (2 to 4 years). However, a higher prevalence of the influenza virus occurred in children to adolescence (5 to ≤ 18years) and adults (19–49 years) age categories. It is known that young children including infants and toddlers suffer a heavy burden of ILI, their complications, but this age group is more likely to seek medical care than school or work-aged populations because of the close health attention they receive from the parents/guardians. Therefore, this age group referred to as < 5-year-olds have been over-represented in this study and several previous studies [18–20]. In this study, it was notable that influenza viruses were more prevalent in adult ages. This is not surprising since this age group is recognized to carry more risk factors and other chronic health problems as well as more frequent interactions. This predisposes the adult age population to a range of respiratory infections [21, 22] Thus, due to the predisposing factors, it was possible to capture the adult age population in ILI surveillance programs. Usually, healthy adults with ILI are less likely to seek medical care unless they get severely ill. The school-aged group is at a higher risk of getting infected with influenza viruses [23, 24]. This agreed with our study findings which indicated a high prevalence (24%) of influenza among participants who attended school. In other occupations which included student, military, and other professions, we found a substantial (33%) influenza virus infections. These findings are in agreement with what has been reported elsewhere in militaries, travelers, caregivers, and other people in correctional facilities [25–28].

Many studies have demonstrated interspecies transmission of novel influenza strains in pigs and birds. However, in this study, we were unable to show any interspecies transmission between humans and the various animal species including pigs, birds, and cats. In this report, the prevalence of influenza among those who had household exposure to birds or pigs was similar to or lower than that of those who were not exposed. While this lack of association could be due to factors such as socioeconomic or demographical status, it is more likely that an interspecies transmission is a rare event that is therefore difficult to capture in surveillance studies with a similar study design as ours.

The descriptions of the epidemiology of the influenza viruses in Kenya in this study complement the results of a similar study performed by the CDC during the same period [13]. Our surveillance program was able to not only provide useful data about the routine circulation of influenza viruses but also contributed to responding to influenza outbreaks that occurred in Kenya and the East African region during this period. During the influenza pandemic outbreak in 2009, this surveillance program acted as the *de facto* testing mechanism for Kenya as well as the Republics of Seychelles and Somalia [27]. By leveraging the capacity built-in support of human influenza sentinel surveillance, our program supported the rapid diagnosis and response to the A/H1N1/pdm 2009 influenza pandemic within the region. This is consistent with its objectives and demonstrates the value of these surveillance efforts to public health in Kenya, and globally as well as for both the Kenya Defense Force and the United States military.

Our study may have suffered from several shortfalls. These comprise selection bias in including a high proportion of ILI among children under 5 years old (infants and toddlers), which results in a lower prevalence of infection and limited ability to detect associations in older age groups including senior adult (>50 years). In addition, the study was based on health care facilities at the district level. Due to this higher level of healthcare stratum, it may have included patients who were sicker than the average patient with ILI, resulting in an overestimation of the influenza virus prevalence. Although the sites were selected to provide a thorough representation of the different geography, ecology, and populations of Kenya, there may be other areas for which these estimates may not be generalizable such as borders, refugee settings, or areas that could not be accessed due to security reasons. Such populations may be at higher risk for both high transmissions of seasonal and emerging influenza strains. Finally, errors in misclassification may have led to an underreporting of the prevalence of influenza infection.

Nevertheless, the strengths of this study are considerable, including the large sample size, broad geographic sampling high-quality sample collection, and processing, well-trained personnel, state-of-the-art equipment and laboratories, and a pre-defined study protocol with comprehensive regulatory support.

## Conclusion

Influenza viruses occur commonly among patients with ILI in Kenya and are prevalent in older children and adult populations. Both occupational and school-based settings showed a higher prevalence of influenza viruses. Influenza viruses circulated year-round in Kenya without regular seasonal patterns.

## Supporting information

S1 Table The crude proportion of influenza virus status by clinical characteristics. (TIF)
