# Supplementary figures and images for: Prevalence and factors influencing the distribution of influenza viruses in Kenya: Seven-year hospital-based surveillance of influenza-like illness (2007–2013)
Source: PLoS One. 2020 Aug 21;15(8):e0237857. doi: 10.1371/journal.pone.0237857 (PMC7446924; doi:10.1371/journal.pone.0237857)

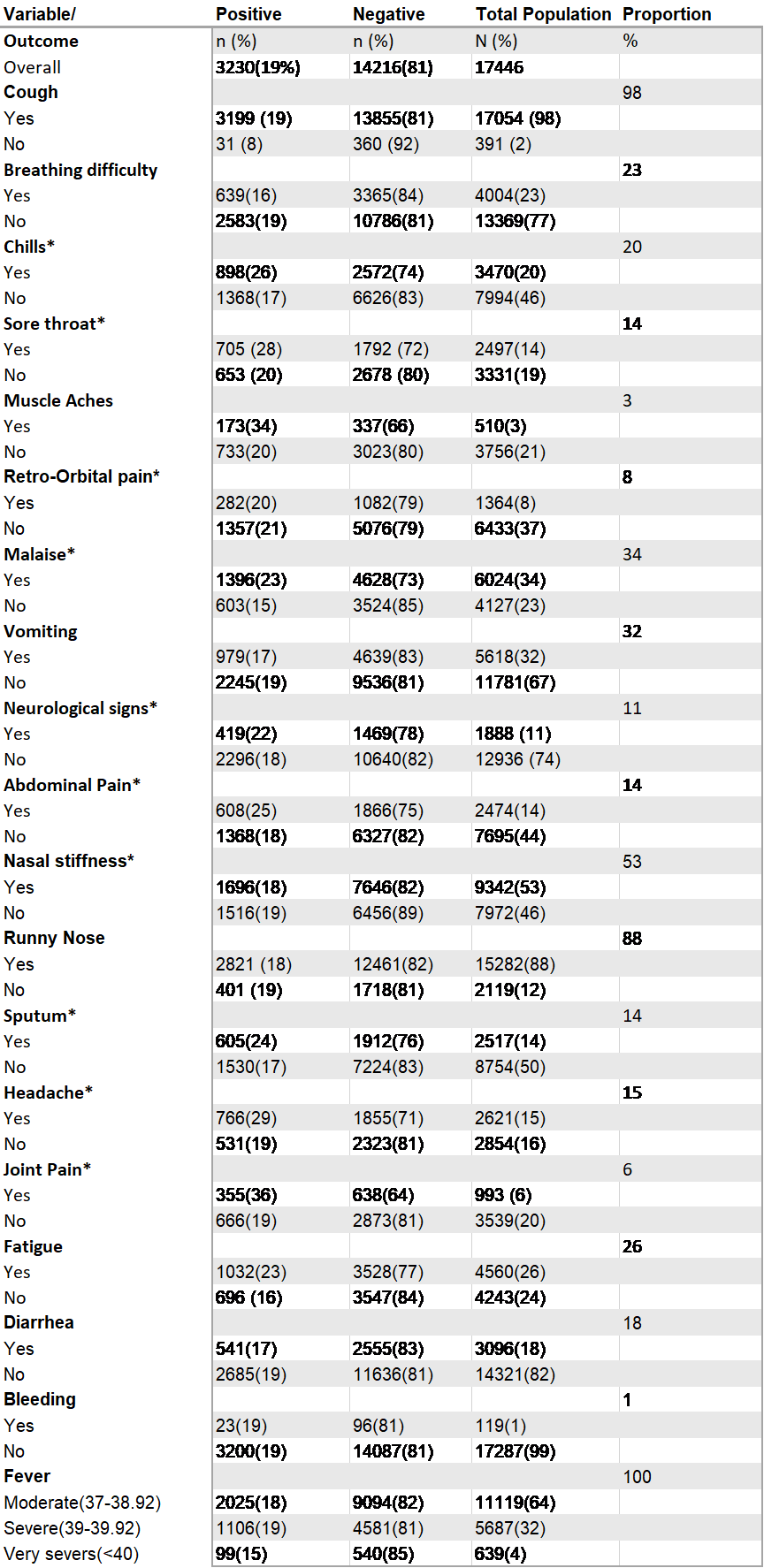

Supplement: S1 Table — (TIF) [file pone.0237857.s001.tif]
